# Experimental malaria: the *in vitro* and *in vivo* blood pressure paradox

**DOI:** 10.5830/CVJA-2011-059

**Published:** 2012-03

**Authors:** CR Nwokocha, DU Owu, IO Ajayi, AB Ebeigbe, MI Nwokocha

**Affiliations:** Department of Basic Medical Sciences, University of the West Indies, Kingston, Jamaica; Department of Basic Medical Sciences, University of the West Indies, Kingston, Jamaica; Department of Physiology, University of Benin, Benin City, Nigeria; Department of Physiology, University of Benin, Benin City, Nigeria; Kingston Public Hospital, Kingston Jamaica

**Keywords:** blood pressure, blood vessel, malaria, vascular reactivity

## Abstract

**Objective:**

Malaria causes more deaths worldwide than any other parasitic disease. Many aspects of the biology that governs the pathogenesis of this parasite are still unclear. Therefore insight into the complexity of the pathogenesis of malaria is vital to understand the disease, particularly as it relates to blood pressure.

**Methods:**

*In vivo* and *in vitro* experimental models were used for this study. In the *in vivo* study, mean arterial pressure, pulse rates and heart rates were recorded by cannulation of the carotid artery of rats. In the *in vitro* study, ring preparations of blood vessels from the rat aorta were studied using standard organ bath techniques. Dose–response curves for phenylepherine (PE)- and acetylcholine (Ach)-induced relaxation were constructed for rings pre-contracted with PE.

**Results:**

Our results showed a significant (*p* < 0.05) reduction in the mean arterial pressure and pulse rates, while the heart rates remained unaltered in rats with malaria parasites, compared with the controls. Incubation of rat aortic rings with parasitised blood resulted in a significant (*p* < 0.05) increase in maximum contractile response to phenylephrine in the rat aortic rings but there was no effect on the baseline. The dose–response curve showed a significant (*p* < 0.05) leftward shift following the addition of parasitised blood and the EC_70_ (M) values increased from 7 × 10^-7^ to 5 × 10^-6^ M. Following exposure to parasitised blood, the magnitude of Ach-induced relaxation responses reduced significantly (*p* < 0.05) from 73 ± 3.6 to 24.75 ± 7.25% in the rat aortic rings.

**Conclusions:**

The results suggest that malaria parasitaemia caused *in vivo* reduction in blood pressure, and enhanced the responses to contractile agents and reduced relaxation responses to acetylcholine *in vitro*. This appears to be a paradox but is explainable by the complex cardiovascular control mechanisms *in vivo*. This may be independent of direct action on vascular smooth muscle.

## Abstract

Malaria causes more deaths worldwide than any other parasitic disease and it is responsible for an estimated 1.5 to 2.7 million deaths annually.^1^ Many aspects of the molecular biology, immunology and epidemiology that govern the pathogenesis of this parasite are still unclear and such insight into the complexity of malarial pathogenesis is vital to understand the disease.

The capacity of *Plasmodium falciparum* to cause severe and fatal disease is believed to be in part due to its ability to sequester in post-capillary venules. Severe *falciparum* malaria is associated with tissue ischaemia related to the cyto-adherence of parasitised erythrocytes to the microvascular endothelium, and reduced levels of nitric oxide (NO) and its precursor, L-arginine.^2^ Malaria has been reported to produce alterations in cardiovascular function.^3^,^4^

Reports in the literature are conflicting; whereas some workers reported a fall in blood pressure (BP) in *falciparum* malaria,^5^-^8^ others have associated it with hypertension and severe intracranial hypertension.^9^,^10^ An increase in cardiac output and systolic right ventricular pressure but reduced heart rate, total peripheral vascular resistance and mean arterial blood pressure have been reported with rising parasitaemia.^3^ Malaria is associated with significant lengthening of the QT interval, which could predispose to potentially lethal polymorphic malignant ventricular tachyarrhythmias.^11^,^12^

The release of haemoglobin (Hb) through intravascular haemolysis, which is then able to scavenge endothelium-derived NO 600-fold faster than erythrocytic haemoglobin, is a central pathophysiological event leading to vascular complications,^13^-^19^ and may contribute to pulmonary arterial hypertension, peripheral vasculopathy and stroke. This is because NO plays a major role in vascular homeostasis and has been shown to be a critical regulator of basal and stress-mediated smooth muscle relaxation, vasomotor tone, endothelial adhesion molecule expression, platelet activation and aggregation.^13^-^15^

Parasitised red cells adhere to constitutive and cytokine-inducible receptors on the microvascular endothelium, resulting in sequestration, vascular obstruction, impaired perfusion, endothelial inflammation and damage.^20^-^24^ They also contribute to the synthesis and release of cytokines and even neurotransmitters,^21^-^23^ the impaired cerebral synthesis of serotonin, dopamine, histamine and norepinephrine,^24^,^25^ and endothelial cell activation.^26^ All these further compound the situation, leading to local metabolic derangements.^5^,^26^,^27^

With increasing sensitivity to vasoconstrictors, vascular resistance is expected to increase, leading to an elevation in blood pressure. The role of cardiac dysfunction in the pathogenesis of severe malaria remains unknown or relatively confusing. The aim of this study was to evaluate, using *in vitro* and *in vivo* methods, the effects of experimental malaria on blood pressure mechanisms, with a view to further understanding its role with regard to blood pressure changes.

## Methods

Five seven-week-old male Wistar rats weighing 150–180 g and six Swiss mice weighing 30 g were obtained and kept at the animal house of the Faculty for the study. The animals were kept at a room temperature of 27 ± 2°C with 12-h light/dark cycles. They were fed with standard rat food and water *ad libitum*. Approval for the study was sought and obtained from the Faculty Ethics and Animal Regulations Committee.

The Swiss mice were used in the induction, maintenance and preservation of the malaria parasite model *(Plasmodium berghei)*, which cannot be maintained in the Wistar rats used for the vascular tissue studies.^28^-^31^

Parasitaemia was maintained in the mice, using *Plasmodium berghei* for the animal model. Briefly, parasitic infection was induced by intraperitoneal injection of 4 × 10^6^ (0.4 ml of parasitised blood in phosphate-buffered saline) *Plasmodium berghei* parasites. Development of parasitaemia in the infected mice was monitored by microscopic examination of a blood film (Giemsastained thin blood films) from the infected mice. On the fourth day post-inoculation, blood pressure and heart rates were measured in the infected and control mice.

All mice were anaesthetised by intraperitoneal injection of sodium thiopentone (50 mg/kg body weight). A polyethylene catheter was inserted into the right jugular vein and another into the left carotid aorta and connected to a pressure transducer (Statham P23XL) and Ugo Basile Polygraph (Model 7050, Varese, Italy) for blood pressure and heart rate (HR) measurements. Heparin (500 IU/kg) (Upjohn) was injected to prevent intravascular blood clotting.

The animals were allowed to stabilise for at least 30 min before recording. The blood pressure was recorded at a chart speed of 10 mm/s and the heart rate was measured by increasing the chart speed on the machine to 50 mm/min. The mean arterial pressure (MAP) was calculated as the sum of the diastolic pressure and 1/3 pulse pressure.

The thoracic aorta of the rats was rapidly dissected out and placed in ice-cold, oxygenated, modified physiological saline solution (PSS) with the following composition (mM): NaCl 119, KCl 4.7, CaCl_2_ 2.5, MgSO_4_・ H_2_O 1.2, KH_2_PO_4_ 1.2, NaHCO_3_ 24.9, and glucose 11.1, pH 7.4. It was then cleaned of loosely adhering fat and connective tissue and cut into ~2-mm rings. Each aortic ring was suspended in an organ bath containing 20 ml of well-oxygenated (95% O_2_, 5% CO_2_) PSS at 37°C. The rings were allowed 90 min to equilibrate before the commencement of the various protocols.

Force generation was monitored by means of an isometric transducer (Grass model FT.03 isometric transducer) connected to a Grass multichannel polygraph (Model 79D, Grass, Quincy, MA, USA). The resting tension in the aortic rings was adjusted to 1.0 g, which was found to be the optimal tension for inducing a maximal contraction in preliminary experiments. The aortic strips were first contracted with 80 mM KCl and this response was taken as 100%.

Contractile responses were each expressed as a percentage of the contraction previously induced by 80 mM KCl. Dose–response tests to phenylephrine were carried out by cumulative addition of the agonist to the bath in the presence or absence of parasitised blood pre-incubated for 10 min.

The relaxation responses to acetylcholine were assessed cumulatively in rings pre-contracted with 10^-6^ M (EC_70_) phenylephrine (PE) in the presence or absence of parasitised blood. The magnitude of relaxation was compared with the pre-contraction induced by phenylephrine.^32^

## Statistical analysis

Results are presented as means ± SEM and comparison of the means was done using the Student’s *t*-test. A *p*-value < 0.05 was considered statistically significant. Contractile responses are expressed as percentage (%) of maximal response to 80 mM KCl. The concentration–response curves for acetylcholine were constructed using computer software from Origin™ 5.0 (Microcal Software Inc, Northampton, USA), and EC_50_ and EC_70_ values (concentrations producing 50 and 70% of maximum responses) were determined graphically.

## Results

**Fig. 1:**
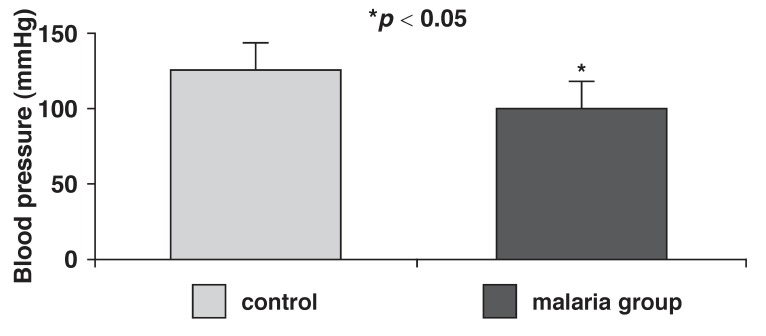
Mean arterial pressure in control and malaria-parasitised rats.

**Fig. 2:**
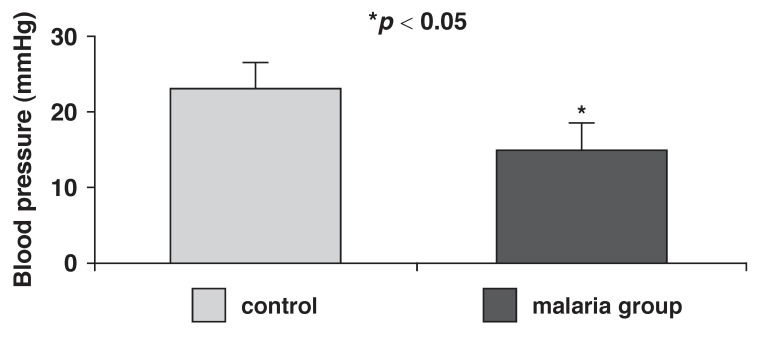
Pulse pressure in control and malaria-parasitised rats.

**Fig. 3:**
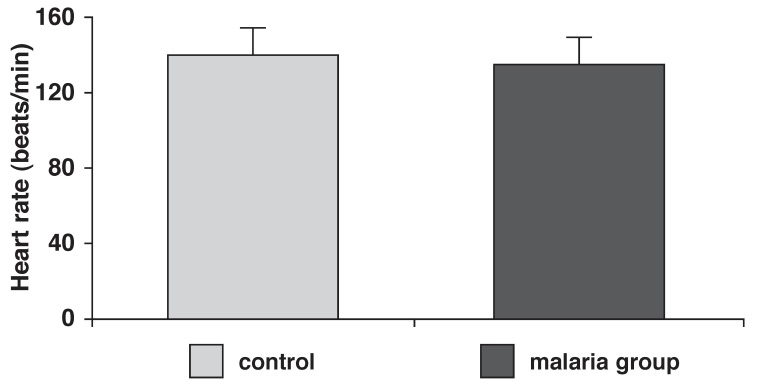
Heart rate in control and malaria-parasitised rats.

The arterial blood pressure, pulse pressure and heart rates of control and malaria-parasitised rats are presented in Fig. 1. Mean arterial pressure was significantly (*p* < 0.05) reduced in the parasitised (100 ± 12 mmHg) rats when compared with controls (125 ± 10 mmHg). Pulse pressure was also significantly (*p* < 0.05) reduced in the parasitised rats (15 ± 5 mmHg), compared with controls (23 ± 3 mmHg) (Fig. 2). Malaria parasitaemia did not significantly alter heart rate (Fig. 3).

**Fig. 4:**
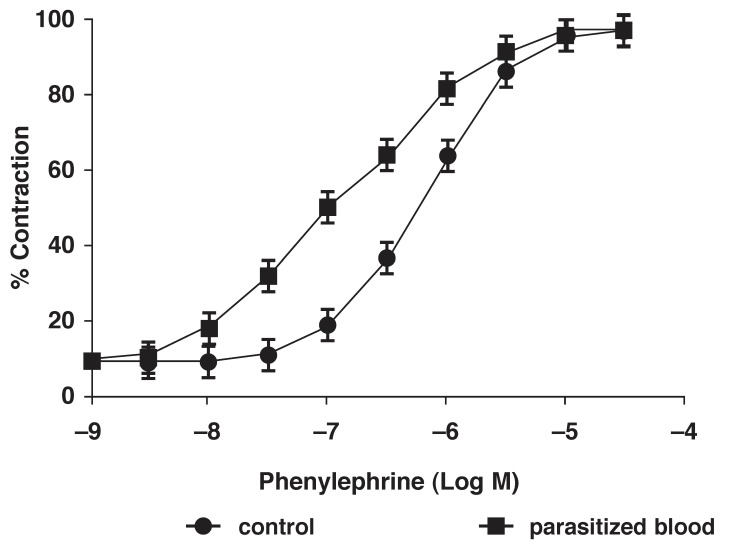
Influence of parasitaemia on phenylephrine concentration response curves in aortic rings. Data are means ± SE (*n* = 6)

The dose–response curves for phenylephrine are presented in Fig. 4. Parasitaemia resulted in a significant (*p* < 0.05) enhancement (leftward shift) of the phenylephrine dose–response curve. The mean EC_70_ values for phenylephrine contractions in the various ring preparations were 7 × 10^-7^ M for the control and 5 × 10^-6^ M for the tissues exposed to parasitised blood. Incubation with parasitised blood alone did not affect the resting tension in the rings studied, and did not cause the relaxation of the phenylephrine pre-contracted rings when added to the organ baths (not shown).

**Fig. 5:**
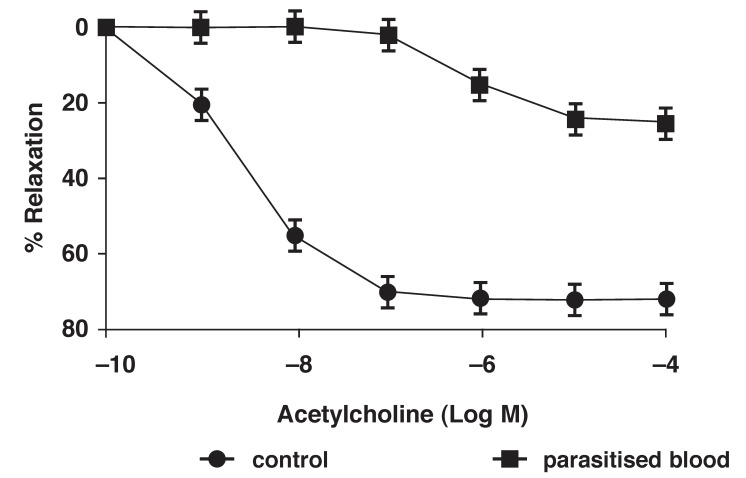
Ach-induced relaxations of aorta following exposure to parasitised blood. Data are means ± SE of tissues (*n* = 6)

Endothelium-dependent acetylcholine-induced relaxation responses were examined in the phenylephrine (10^-7^ M) pre-contracted rings. Parasitaemia significantly (*p* < 0.05) attenuated acetylcholine-induced relaxation of the aortic rings following exposure to parasitised blood (Fig. 5).

## Discussion

In the *in vivo* studies, we observed that the mean arterial pressure and pulse rates were significantly reduced in the parasitised animals, while the heart rates were not altered. This is partly in agreement with Shida,^3^ who reported a reduced mean arterial blood pressure and heart rate.

There was no decrease in heart rates in our parasitised group, compared with the controls and hence the hypotension observed cannot be attributed to bradycardia. The hypotension can also not be explained as an autonomic (sympathetic) activity as there was also a reduction in the pulse rates. This decrease in blood pressure could have been associated with impaired cerebral synthesis of serotonin, dopamine and norepinephrine, enhanced production of histamine,^24^, ^25^ and reduced levels of NO and its precursor, l-arginine, which may lead to vasodilation *in vivo*.

In the *Plasmodium berghei* ANKA (PbA) murine model of Cabrales *et al.*, cerebral malaria pathogenesis was associated with low nitric oxide (NO) bioavailability and brain microcirculatory complications, with a marked decrease in cerebral blood flow, vasoconstriction, vascular plugging by adherent cells, and haemorrhage.^33^

The tone of blood vessels is determined by their responsiveness to contracting agonists. In the present study, incubation with parasitised blood resulted in a significant increase in maximum contractile response to phenylephrine in the rat aortic rings. Analysis of the whole dose–response curve showed a significant leftward shift of the curve following the addition of parasitised blood. There was increased sensitivity to phenylephrine in the aortic rings exposed to parasitised blood. We also observed a significant reduction in the magnitude of Ach-induced relaxation following exposure to parasitised blood.

Shida *et al.*^3^ had earlier reported a decline in the total peripheral vascular resistance, but our observations were an enhanced vascular reactivity *in vitro.* This increased vascular reactivity could lead to elevated blood pressure. The enhanced contractions with parasitised blood could have been due to the non-specific immune inflammatory response of the host to the malaria parasite, with the release of various mediators into the blood stream, and local synthesis of cytokines or even neurotransmitters^21^-^23^ associated with endothelial inflammation and damage.^34^-^36^

Previous studies have reported an overall reduction in nitric oxide availability with malaria infection.^14^-^16^ Increased cell-free haemoglobin and plasma arginase^13^,^15^-^19^ would have caused reduced nitric oxide availability, which could possibly have affected the relaxation responses to acetylcholine and also enhanced the phenylephrine-induced contraction.

The enhanced production of histamine and NO^24^ would be expected to cause vasodilation, while local cytokine production^25^ would be expected to cause an increase in vascular tone. Incubation of the aorta with malaria-parasitised blood did not have any effect on baseline contractions. This suggests that interaction between malaria parasites and the blood vessel wall *(in vitro)* resulted in functional changes in the contractile state of the vascular smooth muscles, possibly through the release of vaso-active agents from both the red cells and vascular endothelium.^37^

With malaria infection, the vasculature exhibits not only an impaired vasorelaxant response but also a markedly exaggerated vasoconstrictive response. This enhanced sensitivity of the vasculature to adrenergic activation may therefore be one of the numerous pathophysiological mechanisms leading to end-organ damage of some organs following malaria infection.

The reduction in blood pressure *(in vivo)* induced by malaria parasitaemia is a paradox, with the *in vitro* results showing enhanced response to contractile agents and reduced relaxation response to acetylcholine. Since the endothelium-dependent relaxation was attenuated in malaria, there is a need for further studies on nitric oxide synthase expression in the blood vessels with malaria parasitaemia. The augmented vascular responses we observed may be explained by differences in receptor and protein expression. A study of such expression could provide more insight on how they are able to modulate cardiovascular control mechanisms.

## Conclusion

Malarial infection caused a reduction in blood pressure without affecting the heart rate *in vivo*, whereas there was an enhanced contractile response and attenuated vasorelaxation *in vitro*. This can be explained by the complex cardiovascular control mechanisms *in vivo*, which are independent of direct action on vascular smooth muscle.
